# Patient-specific effects of metformin on the hepatic metabolism in adolescents with metabolic dysfunction-associated steatotic liver disease (MASLD)

**DOI:** 10.1007/s00109-025-02551-y

**Published:** 2025-05-27

**Authors:** Hermann-Georg Holzhütter, Christian A. Hudert, Nikolaus Berndt

**Affiliations:** 1 Charité – Universitätsmedizin Berlin, corporate member of Freie Universität Berlin and Humboldt-Universität zu Berlin, Institute of Biochemistry, Charitéplatz 1, 10117 Berlin, Germany; 2 Charité-Universitätsmedizin Berlin, corporate Member of Freie Universität Berlin and Humboldt-Universität zu Berlin, Department of Pediatric Gastroenterology, Nephrology and Metabolic Diseases, Berlin, Germany; 3 German Institute of Human Nutrition Potsdam-Rehbruecke (DIfE), Department of Molecular Toxicology, Nuthetal, Germany; 4 Charité – Universitätsmedizin Berlin, corporate member of Freie Universität Berlin and Humboldt-Universität zu Berlin, Department of Radiology, Berlin, Germany

**Keywords:** Metformin, Liver metabolism, Computational model, Pediatrics, MASLD

## Abstract

Metformin is a commonly prescribed antidiabetic drug that inhibits hepatic glucose production (HGP). Recent studies examining the use of metformin for the treatment of children with metabolic dysfunction-associated steatotic liver disease (MASLD) showed controversial results. To evaluate the patient-specific impact of metformin on hepatic glucose, lipid, amino acid, and energy metabolism in a cohort of 70 paediatric patients with biopsy-proven MASH. We parametrized our mathematical model HEPATOKIN1 of liver metabolism with patient-specific proteomics data of liver enzyme abundances and simulated metformin-induced diurnal changes of a large panel of metabolic functions. On average, a single dose (250 mg) of metformin reduced diurnal HGP by 19%. Based on a Z-score of 1, 15% of patients were classified as low responders or high responders. During elevated metformin plasma levels within four after metformin ingestion, energy metabolism, cytosolic and mitochondrial redox potential, urea synthesis and ketone body synthesis were reduced by 10–30%, but averaged over 24 h, these metabolic side effects were not significant. In particular, there was no significant impact of metformin on hepatic fat storage. Baseline lactate and insulin activity at 90 min after glucose challenge (OGTT) correlated significantly with the reduction in HGP and may serve as predictors of effective therapy. On a daily average, metformin selectively affects hepatic glucose production, glycogen storage and lactate uptake, while numerous other metabolic functions are significantly altered only for several hours after administration of the drug. Our method provides a patient-specific analysis of the potential effects of metformin therapy on central hepatic metabolism and may therefore help guide the physician's therapeutic decision.

## Introduction

### The medical importance of metformin

The anti-hyperglycaemic drug metformin is considered a first-line treatment for type 2 diabetes mellitus (T2DM) [1], often prescribed alongside lifestyle changes, such as diet and exercise. Metformin may also be prescribed for individuals with prediabetes, a condition where blood sugar levels are higher than normal but not yet high enough to be diagnosed as diabetes. Metformin is sometimes used off-label to treat polycystic ovary syndrome where it can help to improve insulin sensitivity, regulate menstrual cycles, and promote ovulation [2]. In some cases of gestational diabetes, metformin may be used to help control blood sugar levels. Metformin may also be associated with modest weight loss in some individuals with T2DM or prediabetes [3]. Overall, metformin plays a crucial role in the management of T2DM and related conditions by helping to control blood sugar levels and improve insulin sensitivity. Metformin is sometimes prescribed to children and adolescents, typically for the treatment of T2DM [4]. However, its use in paediatric populations is generally more limited. The American Diabetes Association recommends considering metformin therapy for children and adolescents with T2DM, particularly in those who have failed to achieve target glycaemic control through lifestyle interventions alone. Recent studies examining the use of metformin for the treatment of children with metabolic dysfunction-associated steatotic liver disease (MASLD) showed controversial results [4, 5].

### Impact of metformin on liver metabolism

There is a consensus that the glucose-lowering effect in patients with T2DM is mediated mainly by inhibition of hepatic gluconeogenesis. However, despite decades of research, the mechanism by which metformin inhibits this process is still controversial. Widely studied mechanisms of metformin action, such as complex I inhibition (reviewed by Fontaine in 2018 [6]) leading to 5'AMP-activated protein kinase (AMPK) activation, have only been observed in the context of supra-pharmacological (> 1 mM) metformin concentrations, which do not occur in the clinical setting. Moreover, animal models suggest that AMPK is dispensable for the anti-hyperglycaemic effects of metformin, as animals with liver-specific genetic deletion of AMPK or the upstream kinase ‘liver kinase B1’ are still able to reduce hepatic gluconeogenesis in response to metformin [7]. Based on the observation that metformin alters cellular redox balance, a redox-dependent mechanism of action has been described by several groups [8–10]: Metformin inhibits the enzyme mitochondrial glycerophosphate dehydrogenase (GPD2), which together with the cytosolic glycerophosphate dehydrogenase (GPD1) catalyses the shuttle of redox equivalents from cytosolic to mitochondrial NADH under reduction of glycerophosphate to dihydroxyacetone phosphate, an intermediate of the gluconeogenetic pathway. Reduced supply of dihydroxyacetone phosphate from glycerol and lowering of the ATP level due to reduced transfer of redox equivalents into the mitochondrial matrix result in an inhibition of gluconeogenesis. On top, the elevated NADH/NAD^+^ cytosolic ratio inhibits the enzyme lactate dehydrogenase and thus the conversion of lactate to the gluconeogenetic precursor pyruvate. A possible side effect of this mode of action consists in a reduced recycling of lactate to glucose (Cori cycle) with the risk of lactate acidosis [11]. Whether metformin can significantly alter other metabolic functions of the liver, at least transiently, is not yet known. In particular, controversial results have been obtained regarding the ability of to reduce hepatic steatosis in vitro and in vivo [12].metformin.

### Assessing the effectiveness of metformin treatment

The therapeutic response of metformin treatment shows great inter-individual variability. In a clinical study assessing the glycaemic response of metformin in patients with T2DM, about 40% of the 200 patients proved to be non-responders [13]. Genetic variations and differential gene expression may play an important role here [14]. Since the administration of metformin in adolescents is contentious due to possible side effects, a pre-treatment assessment of the expected therapeutic efficacy of the drug would be useful. A promising method for this is the simulation of drug effects with the help of mathematical models, which meanwhile are widely used to support decision-making at all stages of drug development and administration [15].

### Aim of the work

Here, we report the results of an in silico study evaluating the effect of metformin on liver metabolism in adolescents with metabolic-dysfunction associated steatotic liver disease (MASLD), some of them with early-stage diabetes mellitus [16].

## Methods

### Patients

This study was based on clinical and proteomic data from a previous study by Berndt et al. [16]. The study protocol conformed to the guidelines of the Declaration of Helsinki and was approved by the local ethics board (EA2/059/14). Informed consent was obtained from all parents or guardians. Patients were recruited from the paediatric obesity outpatient clinic and paediatric gastroenterology outpatient clinic of the Charité. Blood work including liver function tests was performed as part of standard care and prompted a further workup. Diagnostic tests were obtained for exclusion of α1-antitrypsin deficiency, lysosomal acid lipase deficiency, celiac disease, autoimmune hepatitis, viral hepatitis, active cytomegalovirus, or Epstein-Barr virus infection, and Wilson disease. Children and adolescents aged 8 to 17 years who were overweight or obese and suspected of having MASH and a clinical indication for liver biopsy were evaluated for enrolment in the study. Exclusion criteria were as follows: age of at least 18 years, any concurrent liver disease (e.g. inborn errors of liver metabolism such as defects of the urea cycle or fatty acid oxidation), severe underlying chronic disease (e.g. cardiopulmonary or autoimmune disease), alcohol consumption greater than 20 g per day, and pregnancy.

### Kinetic modelling of hepatic metabolism

Metabolic capacities of the liver were assessed using the kinetic model HEPATOKIN1 of liver metabolism [17] combined with a molecular resolved model of hepatic lipid droplet metabolism [18]. Hormone-dependent regulation of the liver metabolism by reversible enzyme phosphorylation was considered by a phenomenological transfer function as described by Berndt et al*.* [17]. Patient-specific model instantiations were generated based on individual proteomic profiles [16].

A standard plasma profile of nutrients and hormones was used as model input for all patients [17]. The hepatic capacity to produce or utilize any metabolite X that is exchanged with the plasma was evaluated by the net amount X_24_ exchanged during 24 h, i.e.

1 $${X}_{24}=\int\limits_{0}^{24}dt {v}_{x}\left(t\right)$$

v_x_(t) represents the exchange rate of metabolite X given in µmol/g/h. X_24_ > 0 indicates net uptake by the liver, and X_24_ < 0 indicates net release. The storage capacity for glycogen and triacylglycerol (TAG) was assessed by their average liver content. The 24 h—average effect of metformin on the hepatic exchange/storage of metabolite X was assessed by

2 $$\Delta {X}_{24}={X}_{24}^{+met}-{X}_{24}^{-met}$$

#### Metformin dynamics and metabolic action

**Fig.1:**
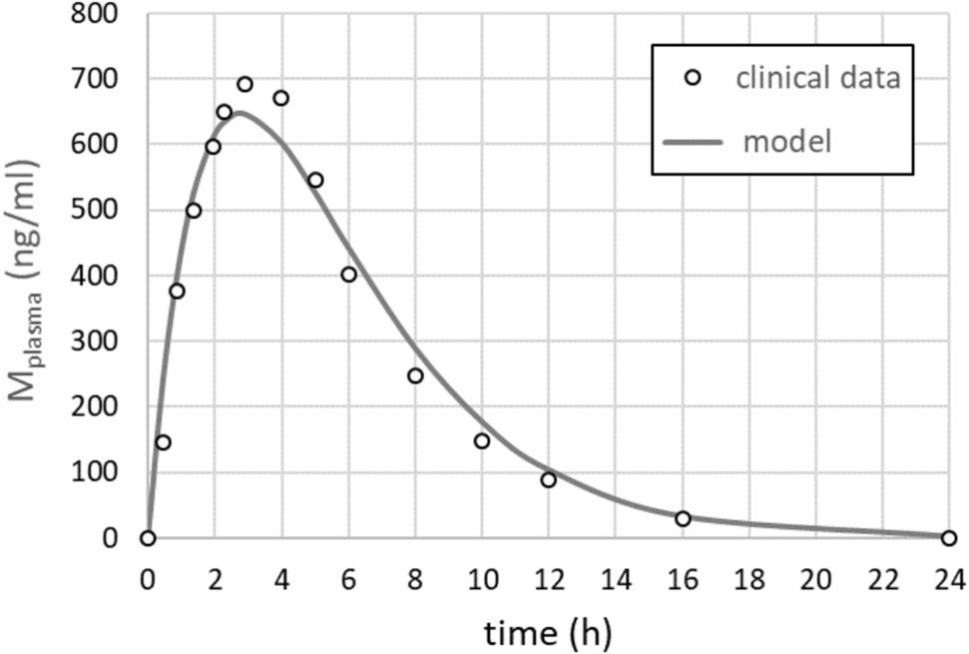
Plasma profile of metformin used in the model simulations

The metformin plasma concentration after ingestion of a single dose of metformin at t = 0 h was modelled by a sum of two exponentials (Fig. 1).

A bi-exponential function ${M}_{plasma}=\frac{{k}_{1}{M}_{0}}{{k}_{2}-{k}_{1}}\left({e}^{-{k}_{1}t}{e}^{-{k}_{2}t}\right)$ was used with parameters k_1_ = k_2_ = 0.36 h^−1^. The parameter M_o_ is proportional to the initial dose of metformin and was chosen to best match the experimental data points taken from Mak et al. [19].

The intra-hepatic concentration of metformin was put equal to the plasma concentration as the equilibration of concentrations, mainly accomplished by the organic cation transporters OCT1 and OCT3 [20], occurs within a short period of about 10 min [21]. Metformin inhibition of complex 1 of the respiratory chain and GPD2 was modelled as non-competitive inhibition with half-saturation constants ${K}_{i}^{complex1}=79mM$ [8] and ${K}_{i}^{GPD2}=55\mu M$ [10].

### Statistics

The association between two arbitrary variables was tested using Spearman’s rank correlation coefficient (R). For p-values < 0.01 the association was considered statistically significant. Student's t-test was used to test whether the difference between the mean values of two independent data sets was statistically significant (*p* < 0.01) or not.

## Results

### Patient characteristics

The cohort of 70 adolescents presented with hypertriglyceridemia (average steatosis score = 2.3) and insulin resistance (average HOMA-IR = 7.0 with HOMA-IR = glucose (mmol/L) x insulin (µIU/mL)/22.5.). The characterization of all patients included (i) anthropometric measures, (ii) histological examination of liver samples, (iii) a comprehensive laboratory analysis including a panel of liver-specific enzymes and complete blood count. On top, metabolic serum parameters including lipid profiles, homeostatic model assessment of IR (HOMA-IR), lactate, pyruvate, and uric acid were assessed (see Table 1).

**Table 1:** *Patient Characteristics* All reported values are expressed as mean ± SD unless indicated otherwise. ALT: alanine aminotranferase; AST: aspartate aminotransferase; BMI-SDS: BMI standard deviation score; GGT: gamma glutamyltransferase; HOMA-IR: homeostasis model assessment for insulin resistance; Values for oral glucose tolerance test include glucose and insulin at time points before (fasting state) and after standardized oral glucose challenge (60 min, 90 min, 120 min). NASH-CRN: nonalcoholic steatohepatitis clinical research network. NAS: NAFLD activity score

Variable	Value (*n* = 70)
Male gender, n (%)	55 (78.6)
Age (years)	14.2 ± 2.2
BMI	34.9 ± 6.4
BMI-SDS	2.8 ± 0.6
Fasting glucose (mg/dl)	87 ± 19
Glucose 60 (mg/dl)	137 ± 35
Glucose 90 (mg/dl)	122 ± 28
Glucose 120 (mg/dl)	114 ± 28
Fasting Insulin (mU/l)	32.4 ± 14.9
Insulin 60 (mU/l)	237.1 ± 145.5
Insulin 90 (mU/l)	205.7 ± 151.5
Insulin 120 (mU/l)	200.1 ± 174.9
Triglycerides (mg/dl)	135 ± 73
Cholesterol (mg/dl)	165 ± 29
HDL (mg/dl)	43 ± 9
LDL (mg/dl)	104 ± 24
HOMA-IR	7.0 ± 3.7
HbA1c (%)	5.3 ± 0.9
ALT (U/l)	108 ± 68
AST (U/l)	63 ± 41
GGT (U/l)	51 ± 36
Steatosis grade (NASH CRN), n (%)	
1	13 (18.6)
2	24 (34.3)
3	33 (47.1)
Fibrosis stage (NASH CRN), n (%)	
0	19 (27.1)
1	18 (25.7)
2	15 (21.4)
3	18 (25.7)
4	0 (00.0)
NAS	3.7 ± 1.5
NAS < 5, n (%)	46 (65.7)
NAS ≥ 5, n (%)	24 (34.3)

**Fig. 2:**
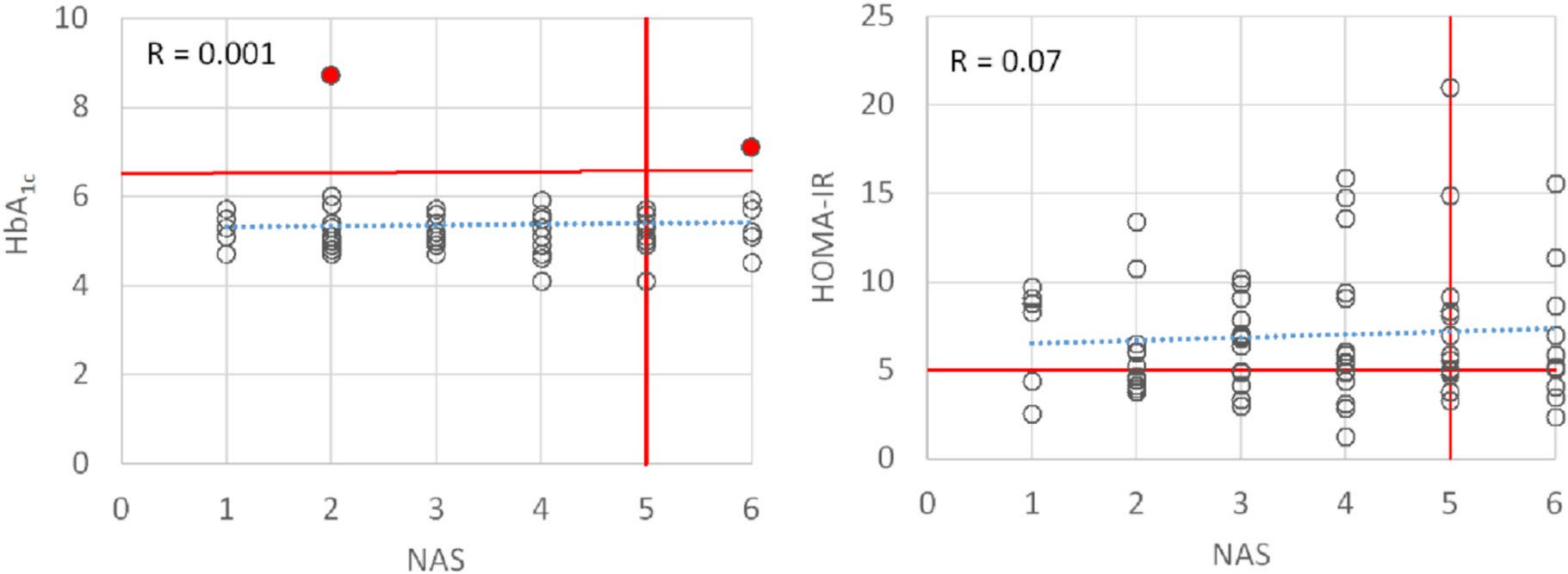
Relationship between severity of MASLD and dysregulation of glucose metabolism. The severity of MASLD was assessed using the NAS score [22] which sums up the individual scores for steatosis (0–3), lobular inflammation (0–3) and ballooning (0–2). Values of NAS > = 5 are considered indicative of the presence of MASH. The parameter HOMA-IR was used to determine the presence of insulin resistance. It is calculated from the product of fasting glucose (mg/dl) and fasting insulin (µU/ml). The threshold value for the presence of IR was set to 5. The presence of type 2 diabetes was evaluated using the parameter HbA1c representing the percentage of haemoglobin glycosylated at the *N*-terminal valine of the ß-chain of HbA1. Values of HbA1c > 6.5% were considered as indication for the presence of diabetes type 2 (T2DM). The threshold values for HOMA-IR, HbA_1c_ and NAS are indicated by the red lines

The mean total plasma cholesterol was near normal, but seven patients showed hypercholesterolemia. Liver histology revealed a broad range of disease severity, including simple steatosis, to severe active steato-hepatitis and a different fibrosis stages range from no to advanced fibrosis, but no evidence of cirrhosis. Portal and lobular inflammation were found in 63.4% and 69% of the patients, respectively. Hepatocyte ballooning indicating severe hepatocellular damage was seen in 48% of the cohort. In addition to the diagnosis of MASLD, the majority of patients also exhibited disturbances in systemic glucose metabolism. Specifically, 68% of patients exhibited pre-diabetic characteristics (HOMA-IR > 5), one patient presented with manifest diabetesT2DM (HbA1c > 6.5%, fasting glucose level G0 > 225 mg/dl). No statistically significant correlation was identified between the severity of insulin resistance and the degree of MASLDNAS (see Fig. 2).

### Model-based assessment of patient-specific metabolic capacities

**Fig. 3:**
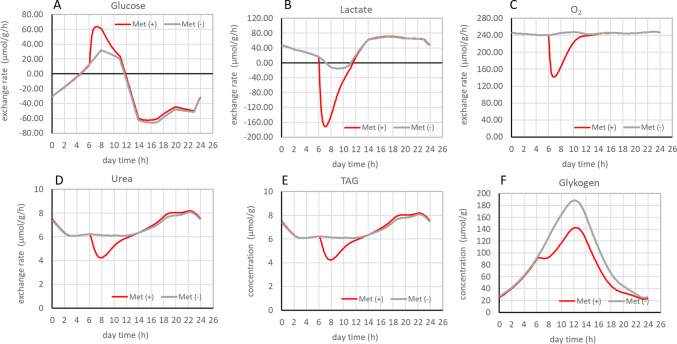
24-h metabolic profile of the liver of a 17-year-old male patient with moderate fibrosis (F2) and presence of MASH (NAS = 5). Exchange fluxes of glucose (**A**), lactate (**B**), oxygen (**C**) and urea (**D**); Cellular concentrations of glycogen (**E**); cytosolic redox potential (**F**). Negative values of exchange rates in A and B indicate net release. The same diurnal plasma profile as in [17] was used for extrahepatic nutrient and hormone concentrations

As described in Methods, we computed for each patient the diurnal metabolic capacities of the liver to exchange metabolites with the plasma and to store TAG and glycogen. These computations were performed in the absence of metformin (control) and in the presence of metformin administrated at 6 h in the morning with a dose of 250 mg yielding the plasma profile shown in Fig. 2. As an example, Fig. 3 shows the 24-h profile of selected metabolic functions of the liver of a 17-year-old male adolescent with moderate fibrosis (F2) and MASH (NAS = 5). In the absence of metformin, the glucose exchange rate is mainly negative, i.e. averaged over 24 h, the liver of this patient acts as a net glucose producer. The 24 h net release of glucose, as quantified by the area under the grey curve (see equn (1)), amounts to Glu_24_ = 437 µmol/g. Under metformin therapy, the glucose uptake rate significantly increases for about 4 h, reaching a maximum of 40 µmol/g/h about 30 min after drug intake. Averaged across 24 h, metformin reduces the net glucose release to −336 µmol/g (= 23% reduction). The lactate exchange varies between net uptake and release but averaged over 24 h, the liver of this patient represents a net lactate consumer with Lac_24_ = 65 µmol/g. Metformin causes a strong increase of the lactate release thus turning the 24 h net uptake of lactate to a net release of 244 µmol/g. When the cellular concentration of metformin reaches its peak value, the uptake rate of O2 is reduced by about 30%, which means a significant restriction of oxidative phosphorylation. Urea production and the glycogen store also become significantly reduced during elevated metformin plasma levels. Consistent with the action of metformin as inhibitor of the glycerophosphate dehydrogenase, the cytosolic NAD/NADH ratio displays a sharp transient drop from values of about 400 down to values of 150.

**Fig. 4:**
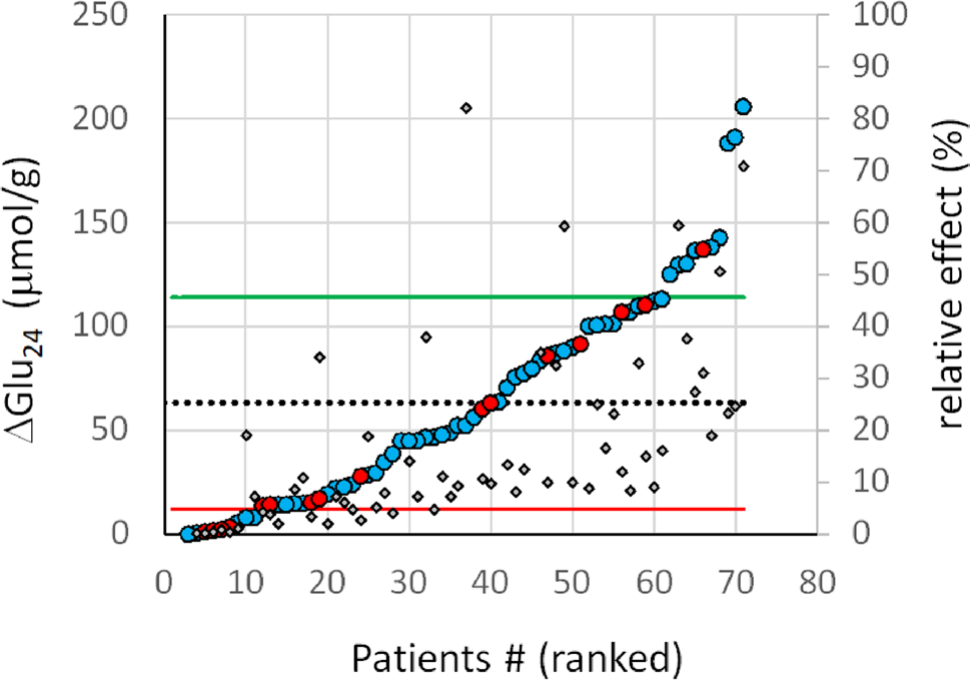
Metformin-induced reduction of diurnal hepatic glucose output ΔGlu_24_ (*N* = 70). Patients were ranked with ascending values of ΔGLU_24_. Circles mark absolute values of ΔGLU_24_ (blue – males, red – females), diamonds mark % relative changes ΔGLU_24_/GLU_24_. The mean value (= 62 µmol/g) is indicated by the dotted line. The mean value ± standard deviation (= 50 µmol/g) were chosen as threshold values (indicated by the green and red line) for the sub-division of patients into 3 response categories: Normal responders = 50 patients (70%): High responders = 10 patients (14%) and low responders = 11 patients (16%). The diamonds indicate the relative effect of metformin given by the ratio $\Delta {Glu}_{24}=\left({Glu}_{24}^{+met}-{Glu}_{24}^{-met}\right)/{Glu}_{24}^{-met}$

Figure 4 depicts the metformin-induced 24 h reduction of the hepatic glucose output in the 70 patients of the paediatric cohort. The mean reduction of HGP was $62\left(\pm 52\right)$ µmol/g. Compared to the glucose output of 329 µmol/g in the absence of metformin, this means an average reduction of 19%. Based on the overall mean ± S.D., the cohort was subdivided into three response classes. The class of low-responders includes 11 patients (16%), whereas 10 patients (14%) are predicted as high-responders. One patient with T2DM showed a metformin effect of ΔGlu_24_ = 23.4 µmol/g/24 h (4.7%) and thus ranked at the lower end of the range of normal responders. Notably, glucose output was actually increased by metformin in two patients who had much higher than average expression of all glycolytic enzymes. The correlation between the absolute and relative effect of metformin was not statistically significant (R = 0.0047; *p* = 0.48), meaning that the individual Glu_24_ value in the absence of metformin is not indicative of the expected effect of the drug. There was no statistically significant difference between mean metformin effect on Glu24 between males (55) and females (16) participants according to the two-sample one-sided t-test (*p* = 0.46).

**Fig. 5:**
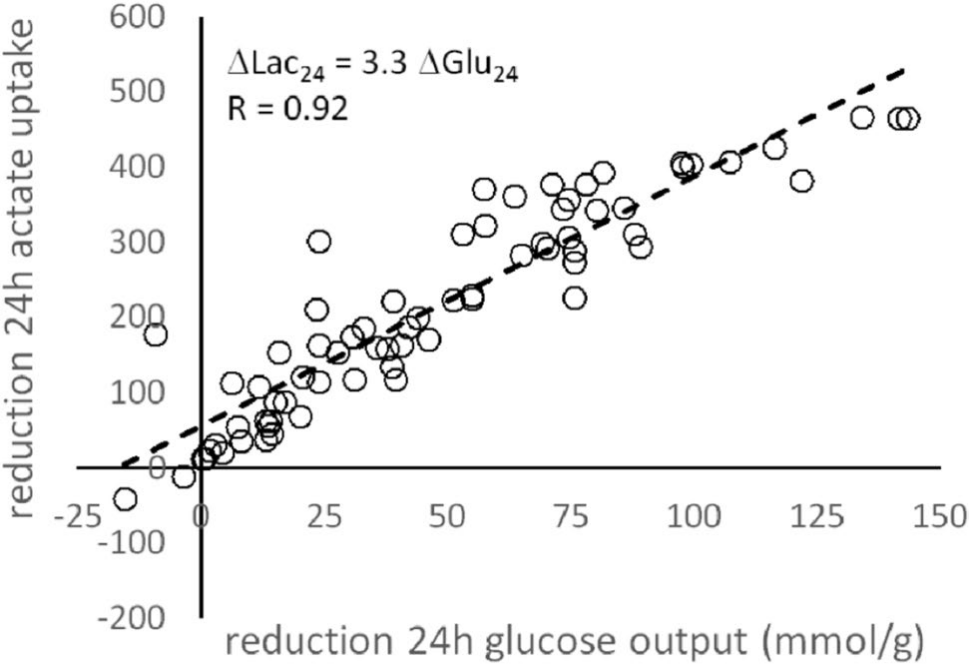
Relationship between metformin-induced diurnal (24 h) reduction of hepatic glucose output (ΔGlu_24_) and diurnal (24 h) lactate release (ΔLac_24_)

As shown in Fig. 5, the reduction of the 24 h glucose production achieved by metformin in the individual patients strongly correlates with the reduction of the hepatic lactate uptake. The slope of the regression line in Fig. 5 indicates that reducing the 24 h glucose output by ΔGlu_24_ = 1 µmol/g is approximately equivalent to reducing the lactate uptake by ΔLac_24_ = 3.3 µmol/g.

Table 2 depicts the mean metformin-dependent changes in the diurnal metabolic capacity of the liver for a panel of physiologically relevant metabolites. We computed the changes for a single dose (250 mg) and a double dose (500 mg). The most significant changes that occurred after a single dose were as follows. Besides a significant reduction in the hepatic glucose output and lactate uptake, a reduction of more than 10% was only found for the peak value (= maximal filling) of glycogen. The largest change (220%) occurred for lactate: metformin transforms the liver from a net lactate consumer (- 99 μmol/g/24 h) to a net lactate producer (119 μmol/g/24 h). The marginal decrease in oxygen uptake and the mitochondrial membrane potential of less than 2% suggest that metformin has little effect on the energy metabolism of the liver when averaged over a 24-h period. However, as the example in Fig. 2 demonstrates, metabolic side effects may be more severe in the first few hours after drug administration compared with the average over a 24-h period. Therefore, we also analysed the changes in the metabolic capacity of the liver during the first four hours after drug intake (6 h-10 h). During this period, the average oxygen consumption of the cohort was reduced by about 35%, and the mitochondrial membrane potential was increased by about 8%. Together with the decline of the cytosolic and mitochondrial redox potentials by about 25% and a reduction of the oxygen uptake by 16%, this indicates a significant transient reduction in energy metabolism, which is manifested in the reduction of the highly energy-dependent urea formation by around 14%.

**Table 2:**
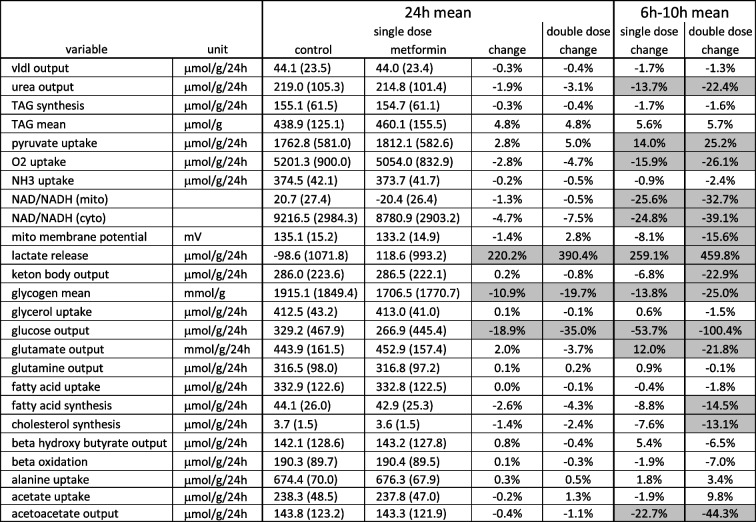
0–24 h and 6–10 h mean metabolic capacity of the liver with and without metformin treatment. Metformin-induced changes larger than 10% are highlighted in grey

With a double dose of metformin, the changes in glucose output and lactate formation also almost doubled, while the changes in all other metabolic functions remained below 10% when averaged over 24 h. For the time window 6–10 h, 14 of the 25 metabolic functions examined were now altered by more than 10%. Particularly striking was the strong reduction in the production of ketone bodies. On average, the change in metabolic functions at double dose compared to the change at single dose was 1.7-fold (24 h) and 1.5-fold (6–10 h), respectively.

We also examined the differences between male and female patients for all of the metabolic effects of metformin examined above. No significant differences were found for any metabolic function. However, given the limited size and disparity of the samples (55/16) of patients, this result cannot be generalized.

**Fig. 6:**
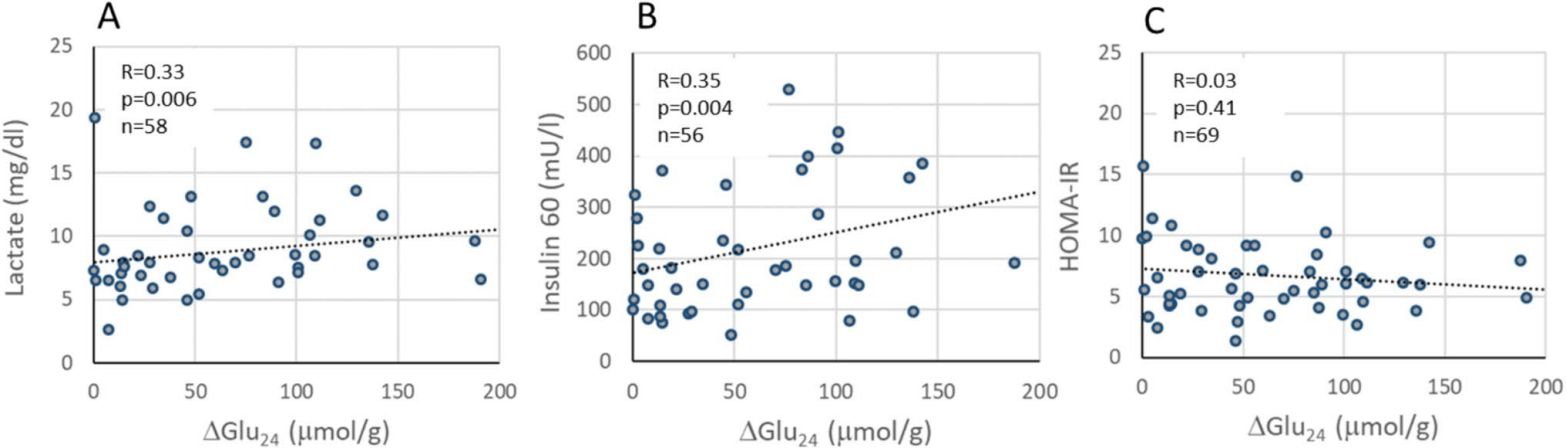
Association of metformin-induced reduction of hepatic glucose output (ΔGlu24) with selected plasma parameters Correlation of ΔGLU24 with plasma lactate (A), plasma insulin activity at 90 min after glucose challenge (Ins90) (B) and HOMA-IR (C)

We also analysed whether some of the routinely measured clinical parameters of our MASLD patients may serve as indicator for the expected response to metformin. For this purpose, we correlated the ΔGlu_24_ values shown in Table 2 with the 50 clinical parameters of which 26 parameters are listed in Table 1. A statistically significant correlation (*p* < 0.01) was only found for two clinical parameters: plasma lactate and plasma insulin activity (Ins60) at t = 60 min after initiation of the oral glucose tolerance test (see Fig. 6A, B). This suggests that these routinely measured clinical parameters may serve as indicators for a sufficient hepatic response to metformin. Interestingly, no association of was found with the HOMA-IR (see Fig. 6C), which, in addition to the Ins60, is an important clinical parameter for the detection of diabetes.

For 45 patients for which values of Ins60 and lactate were available, we performed a contingency analysis using different combinations of the predictor variables lactate and Ins60 (see Table 3). The best discrimination between good responders (ΔGLU_24_ > 62 µmol/g) and poor responders (ΔGLU_24_ < 62 µmol/g) with sensitivity 0.82 and specificity 0.70 was obtained by classifying good responders as having either Ins60 > 400 mU/l or Lac > 8 mg/dl (see Fig. 5C).

**Table 3:**
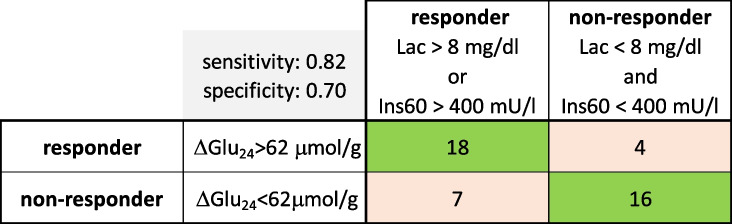
Contingency table comparing the model-based classification of patients as responders (ΔGLU_24_ > 62 µmol/g) and non-responders (ΔGLU_24_ < 62 µmol/g) with the classification based on values of plasma lactate and Ins60 shown (for individual data see Fig. 6A,B). The data set for this analysis included 45 patients for which the plasma values of lactate and insulin(60 min) were available

## Discussion

### Assessment of expected metformin efficacy in reducing the hepatic glucose output

In this work, we predicted the effect of metformin treatment on central hepatic metabolic functions in juvenile MASLD patients using an elaborated and validated kinetic model of human liver metabolism. The credibility of our kinetic model HEPATOKIN1 for model-based predictions of changes in liver metabolism has so far been substantiated by experimental and clinical data in several applications [16, 17, 23–25].

As the treatment of adolescents with metformin is controversial because of possible side effects and limited success [4], a patient-specific in silico assessment of the expected efficacy of metformin treatment may be an important assistance for the physician's final decision to start treatment with metformin. Therefore, our study pursued two objectives: (i) To assess how metformin may impact on central metabolic functions of the liver and (ii) to demonstrate the usefulness of physiologically based metabolic models for the pre-screening of patients concerning the efficacy of a planned drug therapy.

Our analysis showed that the metabolic changes induced by metformin in the liver of adolescent patients with MASLD vary to a large extent. The average reduction in the 24 h hepatic glucose output achieved by a normal single daily dose was 19% in our paediatric cohort. This reduction rate is in line with data reported in the literature. Direct measurement of the hepatic glucose output in nine adult T2DM patients using ^13^C nuclear magnetic resonance spectroscopy yielded an average reduction of 24% after three weeks of metformin treatment, resulting in an average lowering of the fasting plasma glucose level by 30% [26]. Other studies reported a reduction of the HGP between 15 and 30% [27, 28]. Approximately 15% of our patients were predicted to be non-responders, with metformin-induced reductions in liver glucose output being less than 15% of the cohort mean. It should be noted that the large variability of the computed individual responses to metformin is exclusively due to individual differences in the protein levels of metabolic enzymes, which we used to scale the maximal enzyme activities.

### Assessment of expected metformin efficacy in reducing hepatic triglyceride storage

A number of vivo studies in genetically modified mice or dietary models of MASLD rats or mice have demonstrated the effectiveness of intrahepatic lipid reduction by metformin. However, there are also a few contradictory reports (for details, see the review in [12]). The results of our computational study did not provide evidence for a significant lipid-lowering effect of metformin. However, it should be noted that the anti-steatotic effect observed in animal models was attributed to effects of metformin which are not included in our model. These effects include the activation of transcription factors such as TFEB or Stat3, which control autophagy in hepatocytes and thereby lower the abnormal cellular lipid content.

### Temporary impairment of central metabolic liver functions

Due to the short plasma elimination time of metformin, all changes in hepatic metabolism occur only transiently during the first 4–6 h after drug ingestion. When averaged over a full 24-h period, of all the metabolic variables included in our comprehensive model, only the intended reduction of glucose output and the parallel reduction in lactate uptake were significant. However, a significant reduction in hepatic energy metabolism, urea formation, ketone body production and cytosolic/mitochondrial redox potentials was predicted for a time period of about four hours after the administration of metformin. (see Table 1). It is beyond the scope of this study to determine whether these transient reductions in central liver function should lead to dietary and physical activity recommendations for patients on metformin therapy.

### Lactate increase as an indicator of metformin efficacy?

As shown in Fig. 4, an approximately linear relationship was observed between the extent of metformin-induced reduction of the hepatic glucose output and lactate uptake. This fact could potentially be used to determine the efficacy of metformin on hepatic metabolism by measuring under standardized conditions (daytime, overnight fasting) the rise of the plasma lactate level within one hour after administration.

### Metformin’s mode of action

A reliable model-based simulation of metformin effects in liver metabolism requires knowledge of the enzymes affected by the drug and the kinetic parameters relevant to this effect (e.g. IC50 values). Due to its broad use in the treatment of T2DM, there are numerous studies on the molecular basis of metformin, particularly in liver metabolism. It seems certain that the main effect is the inhibition of GPD2, which leads to an inhibition of gluconeogenesis and a shift in the cytosolic redox potential [26]. The originally favoured inhibition of complex 1 of the respiratory chain is now considered less likely due to the high IC50 value, which is far above the plasma concentration of metformin. Nevertheless, this potential mechanism has also been included in the model. In our simulations, we assumed a rapid equilibration of the metformin concentration between plasma, cytosol, and mitochondrial matrix. However, the exchange rate of metformin between cytosol and mitochondrial matrix is not known. Several authors have hypothesized that metformin accumulates in mitochondria (see e.g. [7, 20]. If so, the Nernst equation indicates that for a physiological mitochondrial membrane potential of −180 mV, the thermodynamic equilibrium is reached after a 1,000-fold accumulation of a positively charged molecule if the molecule has one charge. Since metformin is a positively charged molecule and assuming the presence of a still unknown carrier for metformin in the inner membrane, its mitochondrial concentration would reach the millimolar range despite a cytosolic concentration within the micromolar range [5]. In addition, assuming a plasma membrane potential of −36 mV and the absence of kinetic constraints on metformin transporters (OCT and MATE), the cytosolic concentration of metformin would be four times that of plasma. This however seems highly unlikely, as effective concentrations in the mM range would strongly inhibit the respiratory chain complex I (Ki = 0.5 mM) and thus be highly toxic.

### Inferring the metformin response from clinical data

The inclusion of paediatric patients with different degrees of insulin resistance (IR) made it possible to relate the metformin-induced reduction of the hepatic glucose output (ΔGlu_24_) to a large panel of clinical parameters used to assess the severity of MASLD and dysregulation of the glucose metabolism. For only two parameters, baseline plasma lactate and plasma insulin 60 min after oral glucose challenge (Ins60), a significant correlation with ΔGlu_24_ could be established. Increased values of at least one of these two parameters may suggest a higher glucose-lowering effect of metformin. Combining the two parameters by an “or” relation to classify our patients grossly as responders and non-responders yielded a sensitivity of 0.82 and a specificity of 0.70. Hence, plasma lactate values higher than 8 mg/dl or plasma insulin values higher than 400 mU/l at 60 min after a glucose challenge may serve as fairly useful indicators for an above-average hepatic response to metformin.

Interestingly, although both HOMA-IR and Ins60 are clinically accepted parameters for the diagnosis of diabetes and prediabetes, respectively, only Ins60 showed a significant correlation with Glu24. We explain this discrepancy by the fact that increased fasting plasma glucose and plasma insulin levels are caused by IR in various organs (liver, spleen, muscles, adipose tissue), whereas the increase in plasma glucose and thus plasma insulin after glucose challenge is primarily determined by the capacity of the liver to take up most of the glucose during the first passage. This is supported by studies on the diagnostic significance of the glucose tolerance test for the evaluation of metabolic liver function [29].

### Limitations of the approach

A necessary prerequisite for the application of our method is the availability of protein abundance data for liver enzymes, commonly determined in a biopsy sample. It also has to be noted that the presented approach enables only the prediction of short-term metformin effects as drug-induced transcriptional changes [30] would require repeated re-scaling of the model with protein expression data obtained at different time points after the start of the therapy.

Our study operates under the assumption of a uniform metformin distribution across the liver, which may not accurately reflect local variations in microstructure attributable to differences in steatosis, fibrosis, or blood flow. Additionally, liver zonation, which refers to the variability in hepatocyte function based on localization along the liver acinus, could further contribute to heterogeneity, especially considering the zonation of glucose metabolism [25, 31]. Moreover, we analysed the effects of metformin administration at a predefined time point (6 h in the morning). The impact of varying this timing, especially in relation to the diurnal fluctuations in plasma glucose levels, has yet to be explored.

Insulin resistance (IR), i.e. insufficient glucose uptake by cells after insulin stimulation, primarily affects adipose tissue, muscles and liver. For our patients, the relative contribution of the liver to IR was not known and was therefore not considered in the transfer function of the model, which determines the phosphorylation level of key enzymes in dependence of the plasma insulin level.

For all patients, the same diurnal standard plasma profile of metabolites and hormones was used. Very likely, individual differences in the plasma profile of nutrients and hormones will further increase the predicted metabolic variability in metformin response.

As metformin plasma profiles for children and adolescents are not available, we took the 24 h—mean plasma profile determined by Mak et al. [19] as model input for all patients of our cohort. Individual deviations from this standard profile due to differences in the metabolization capacity of the liver (CYP2 C11 und CYP2D1), secretion capacity of the kidney and the total plasma volume will influence the results of our calculations, but a quantitative assessment of such effects was not possible in this study due to the lack of the corresponding data.

Our model-based method allows an estimation of the short-term effect of metformin on the glucose output of the liver while it does not account for systemic effects caused by extrahepatic mechanisms such as its impact on other organs or gut microbiome. For example, a glucose-lowering gut-liver crosstalk has been described which is based on higher plasma lactate and acetate levels [32]. Such effects could, in principle, be incorporated by direct measurement of nutrients and hormones in the blood of the individual patients. Additionally, long-term transcriptional adaptations to metformin treatment were not considered, as they would require dynamic proteomic data across multiple time points. Future work should aim to expand the current model by integrating gut-liver interactions and longitudinal patient data to capture the full spectrum of metformin's metabolic effects.

## Data Availability

The datasets generated during and/or analysed during the current study are not publicly available due to ethical challenges but are available from the corresponding author on reasonable request.

## Abbreviations

AMPK: 5' AMP-activated protein kinase
GPD1: Cytosolic glycerophosphate dehydrogenase
GPD2: Mitochondrial glycerophosphate dehydrogenase
HGP: Hepatic glucose production
HOMA-IR: Homeostasis model assessment insulin resistance
Ins60: Plasma insulin activity 60 min after glucose challenge (oral glucose tolerance test)
NAFLD: Non-alcoholic fatty liver disease
NAS: NAFLD activity score
MASLD: Metabolic dysfunction-associated steatotic liver disease
MASH: Metabolic dysfunction-associated steatohepatitis
T1DM: Type 1 diabetes mellitus
T2DM: Type 2 diabetes mellitus
TAG: Triacylglycerol
